# Robotic-assisted versus open distal pancreatectomy for benign and low-grade malignant pancreatic tumors: a propensity score-matched study

**DOI:** 10.1007/s00464-020-07639-9

**Published:** 2020-08-11

**Authors:** Yuanchi Weng, Jiabin Jin, Zhen Huo, Yusheng Shi, Yu Jiang, Xiaxing Deng, Chenghong Peng, Baiyong Shen

**Affiliations:** Department of General Surgery, Pancreatic Disease Center, Ruijin Hospital, Shanghai Jiao Tong University School of Medicine, No. 197 Ruijin Er Road, Huangpu District, Shanghai, 200025 China

**Keywords:** Robotic-assisted, Distal pancreatectomy, Benign and low-grade malignant tumors, Spleen preservation

## Abstract

**Background:**

This study aimed to compare the short-term outcomes of open and robotic-assisted distal pancreatectomy (ODP and RDP) for benign and low-grade malignant tumors.

**Methods:**

The patients who underwent RDP and ODP for benign or low-grade malignant pancreatic tumors at our center were included. After PSM at a 1:1 ratio, the perioperative variations in the two cohorts were compared.

**Results:**

After 1:1 PSM, 219 cases of RDP and ODP were recorded. The RDP cohort showed advantages in the operative duration [120 (90–150) min vs 175 (130–210) min, *P* < 0.001], estimated blood loss [50 (30–175) ml vs 200 (100–300) ml, *P* < 0.001], spleen preservation rate (63.5% vs 26.5%, *P* < 0.001), infection rate (4.6% vs 12.3%, *P* = 0.006), and gastrointestinal function recovery [3 (2–4) vs. 3 (3–5), *P* = 0.019]. There were no significant differences in postoperative pancreatic fistula, postoperative hemorrhage, and delayed gastric emptying. Multivariate analysis showed that RDP (HR 0.24; 95% CI 0.16–0.36, *P* < 0.001), age (HR 1.02; 95% CI 1.00–1.03, *P* = 0.033), tumor size (HR 1.28; 95% CI 1.17–1.40, *P* < 0.001), pathological inflammatory neoplasm type (HR 5.12; 95% CI 2.22–11.81, *P* < 0.001), and estimated blood loss (HR 1.003; 95% CI 1.001–1.004, *P* < 0.001) were independent predictors of spleen preservation; RDP (HR 0.27; 95% CI 0.17–0.43, *P* < 0.001), age (HR 1.02; 95% CI 1.00–1.03, *P* = 0.022), elevated CA 19–9 level (HR 2.55; 95% CI 1.02–6.39, *P* = 0.046), tumor size (HR 1.44; 95% CI 1.29–1.61, *P* < 0.001), pathological inflammatory neoplasm type (HR 4.48; 95% CI 1.69–11.85, *P* = 0.003), and estimated blood loss (HR 1.003; 95% CI 1.001–1.004, *P* < 0.001) were independent predictors of spleen preservation with the Kimura technique.

**Conclusion:**

RDP has advantages in the operative time, blood loss, spleen preservation, infection rate, and gastrointestinal function recovery over ODP in treating benign and low-grade malignant pancreatic tumors. The robotic-assisted approach was an independent predictor of spleen preservation and use of the Kimura technique.

**Electronic supplementary material:**

The online version of this article (10.1007/s00464-020-07639-9) contains supplementary material, which is available to authorized users.

## Background

Minimally invasive distal pancreatectomy was first applied in 1992 [1] and has become increasingly popular in recent years [2, 3]. Robotic-assisted distal pancreatectomy (RDP) and laparoscopic distal pancreatectomy (LDP), as two kinds of minimally invasive surgery for left-side pancreatic tumors, both showed noninferior short-term outcomes and even oncological outcomes in previous studies [4–6], the newest guideline about minimally invasive pancreas resection, also paid noticeable attention on minimally invasive distal pancreatectomy [7]. While a series of studies have shown the advantages of LDP, a comparison between robotic and open approaches for distal pancreatectomy, especially for benign and low-grade malignant tumors, is lacking.

Patients with benign and low-grade malignant pancreatic tumor patients are always priority candidates for minimally invasive pancreatic surgery. For benign and low-grade malignant tumors in the pancreatic body/tail, LDP has already been proven to have a better postoperative effect than open distal pancreatectomy (ODP).

The surgical treatment of benign and low-grade malignant tumors in the pancreatic body/tail always has the potential for spleen-preserving distal pancreatectomy (SPDP). SPDP is associated with lower rates of postoperative complications [8–10], such as thrombocytosis, embolism, and infection. Splenic vessel-sacrificing (Warshaw technique (WT)) SPDP and splenic vessel-preserving (Kimura technique (KT)) SPDP are two methods applied in SPDP [11, 12]. Although more technically challenging, the KT has a lower risk of splenic infarction and gastric varices, which could occur with the WT [13–15].

Therefore, we designed this study with two aims: to compare the short-term outcomes of ODP and RDP for benign and low-grade malignant tumors and to analyze the factors affecting the spleen preservation rate and the use of KT SPDP.

## Methods

We conducted a retrospective study from a prospective database. This study was performed according to the STROBE guidelines [16]. The study protocol was approved by the Institutional Review Board, Ruijin Hospital, and the requirement for informed consent was waived due to the observational and retrospective nature of the study.

### Patient selection and study design

Between February 2012 and March 2019, all hospitalized patients with a preoperative diagnosis of a benign or low-grade malignant tumor of the pancreatic body/tail at the Pancreatic Surgery Department of Ruijin Hospital were included. Preoperative computed tomography (CT) and magnetic resonance imaging (MRI) scans were routinely performed, and endoscopic ultrasonography was performed for cystic tumors or cases with an unclear diagnosis and surgical indication. The preoperative diagnosis and surgical indication were determined by a multidisciplinary team (MDT) to obtain a more precise and reliable preoperative diagnosis. The MDT consisted of the chief surgeon, first assistant, and at least two professors or associate professors of radiology, and SPDP was performed on an intention-to-treat basis.

An open approach was suggested in a few cases with contraindications of minimally invasive surgery, such as complicated abdominal operation history, severe cardiopulmonary complications, or very old age. In the remaining cases, the selection of RDP and ODP was based on patient preference and acceptance. RDP and ODP were performed by the same group of surgeons in the Pancreatic Surgery Department of Ruijin Hospital, and the team had previous experience in ODP (> 300 cases). To minimize selection bias, we also excluded the initial 40 cases of RDP (from our first case of RDP in 2010 to February 2012) according to previous studies about the learning curve for RDP [17, 18].

Benign and low-grade malignant tumors were defined as pancreatic tumors without oncological side effects treated by SPDP according to the most recent guidelines, including cystic neoplasms, solid pseudopapillary neoplasms, pancreatic neuroendocrine tumors (G1 and G2 without metastasis), inflammatory neoplasms, and other types of pancreatic cancers.

The exclusion criteria included the following: (1) malignant pancreatic tumors, such as pancreatic ductal adenocarcinoma (PDAC), pancreatic adenosquamous carcinoma, pancreatic acinar cell carcinoma, G3 pancreatic neuroendocrine tumors (PNETs), and other pancreatic tumors with malignant biological behavior; and (2) pancreatic neoplasms with suspicion of metastasis, such as G1/G2 PNETs with liver metastasis.

### Surgical protocols

The da Vinci Si Robotic Surgical System (Intuitive Surgical, Sunnyvale, CA, USA) was applied in this study for RDP cases. The procedure for RDP used in this work is similar to that used in a previous article [19] and can be subdivided into the following steps: (1) First, the tumor was exposed, and the possibility of SPDP was evaluated; difficult mobilization of the splenic vessels and high risk of perioperative massive bleeding with the KT were indications for WT SPDP or splenectomy. (2) For KT SPDP, the distal pancreas was mobilized from the splenic vessels, and the branches of splenic vessels were carefully ligated. (3) For the WT SPDP and DP with splenectomy, the pancreas was dissected with the splenic vessels. DP with splenectomy was performed in the following situations: intraoperative signs of splenic ischemia or infarction or a splenic hilum that was technically unable to be transected. (4) The final steps included pathological examination to determine malignant pathological cases that were inappropriate for spleen preservation and to confirm negative margins. In PNETs larger than 2 cm, regional node lymphadenectomy was performed according to the National Comprehensive Cancer Network (NCCN) guidelines. In WT SPDP cases, we reevaluated the blood supply of the spleen. A drainage tube was placed at the surgical site.

### Definitions

Perioperative variables were collected from the hospital’s electronic records system; baseline characteristics included age, sex, body mass index (BMI, kg/m^2^), albumin (ALB) level, previous abdominal surgery history, carbohydrate antigen 19-9 (CA 19-9) level, and American Society of Anesthesiologists Physical Status Classification (ASA score) [20]. We defined portal vein (PV)/superior mesenteric vein (SMV) abutment as PV/SMV compression or lumen narrowing. Tumor size was defined as the longest diameter of the primary tumor or the diameter of the largest tumor in multifocal tumor cases. Regarding intraoperative and postoperative variables, docking time was included in the operative duration in the RDP group, and estimated blood loss was evaluated by the aspirated volume and gauze weight. Postoperative pancreatic fistula (POPF) was defined according to the updated definition of the International Study Group of Pancreatic Fistula [21]; grade A POPF was classified as biochemical leak, and grade B/C was classified as clinically relevant POPF. Postpancreatectomy hemorrhage (PPH) and delayed gastric emptying (DGE) represented complications defined by the International Study Group of Pancreatic Surgery [22, 23]. Infection represented surgical site infection, as defined by the Centers for Disease Control and Prevention (CDC) [24] and diagnosed by positive surgical site pathogen culture, either from the drainage or puncture ascites during the postoperative hospitalization. The complication grade was determined according to the Clavien–Dindo classification [25]. Postoperative interventions, such as vascular embolization, vascular stent placement, percutaneous drainage and endoscopic therapy, were also included in the reoperation rate. Oral intake represented the postoperative returning to liquid diet without gastrointestinal symptoms, regardless of the liquid volume. The discharge criteria included: semifluid diet and able to maintain required caloric intake; no need for intravenous fluids; return to independent mobility or baseline mobility for those with previous mobility deficits. The length of stay (LOS) was defined as the number of postoperative days from the operation to discharge.

### Matching

To minimize the selection bias caused by different characteristics of the patients and tumors, RDP cases were matched to ODP cases using propensity scores. Propensity scores were based on the baseline variables age, sex, BMI, ALB level, previous abdominal surgery history, ASA physical status, CA 19-9 level, and PV/SMV abutment, together with the variations in tumor size, pathological type and tumor location. Propensity score matching (PSM) was performed at a 1:1 ratio, and a caliper width of 0.05 standard deviation (SD) was specified.

### Statistical analysis

Continuous variables with a normal distribution are presented as the mean and SD and were analyzed using Student’s t test or the paired t test. Continuous variables without a normal distribution are presented as the median and interquartile range (IQR), and the Mann–Whitney *U* test was used for analysis between the two groups. Categorical variables are presented as frequencies or percentages and were analyzed by the chi-squared test, Fisher’s exact test or McNemar’s test. Univariate and multivariate logistic regression modeling was performed to analyze the spleen preservation rate and use of the KT. Multivariate analysis was performed after univariate analysis, and the results are presented as hazard ratios (HRs) with 95% confidence intervals (95% CIs). All statistical tests were two-sided, and *P* < 0.05 was considered to indicate a significant difference. Statistical analyses were performed using the statistical package R (The R Foundation; https://www.r-project.org; version 3.4.3).

## Results

### Study group

**Fig. 1:**
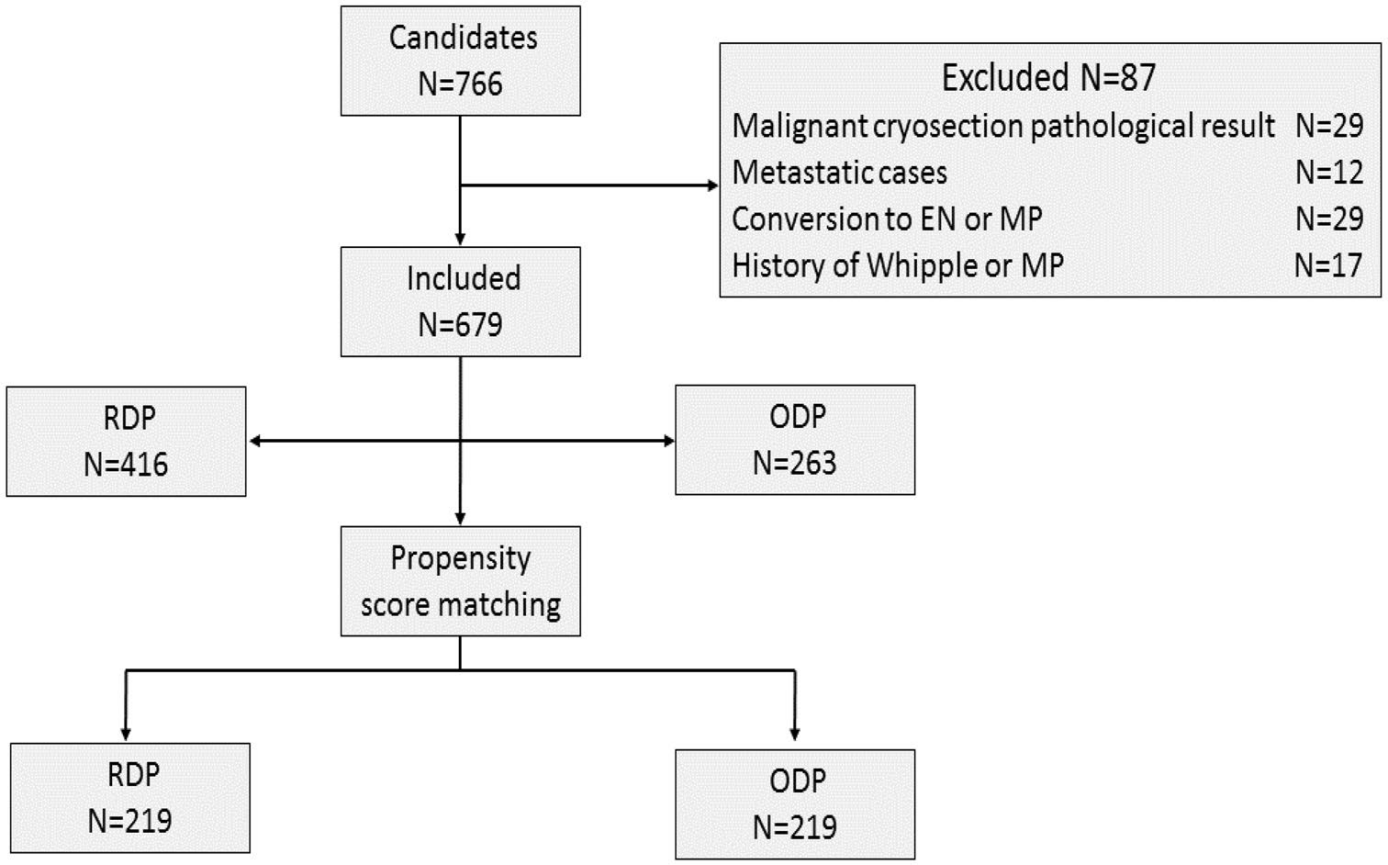
Patient selection flowchart

A total of 766 patients were discussed by the MDT and regarded as candidates with benign or low-grade malignant pancreatic tumor patients for spleen-preserving surgery, and these patients underwent DP on an intention-to-treat basis performed by the same surgical team. Eighty-seven cases were excluded from the final analyzed database for different reasons: (1) intraoperative cryosection pathological examination findings with a malignant component (*N* = 29); (2) metastatic disease, including all PNET cases with distant metastasis (*N* = 12); (3) intraoperative decision to convert to enucleation (EN) or middle pancreatectomy (MP) (*N* = 29); and (4) history of Whipple or middle pancreatectomy (*N* = 17). The remaining 679 patients were included in the final analysis; 416 underwent RDP, and 263 underwent ODP. The study flowchart is shown in Fig. 1. There were three cases (0.7%) of conversion in the RDP group.

### Baseline characteristics

Before matching, the RDP group had a significantly lower age, lower male patient proportion, higher ALB level, lower rate of previous abdominal surgery, and smaller tumor size; significant differences were also found in the ASA physical status and pathological type. After PSM at a ratio of 1:1, 219 patients were included in each group, and the baseline characteristics became equivalent to minimize selection bias for subsequent analysis (Table 1).

**Table 1:** Demographic and baseline characteristics of the study population

	Before propensity score matching			After propensity score matching		
	RDP (*N* = 416)	ODP (*N* = 263)	*P* value	RDP (*N* = 219)	ODP (*N* = 219)	*P* value
Age (years), mean (SD)	47.8 ± 15.5	51.6 ± 15.0	**0.002**	50.4 ± 15.5	51.0 ± 14.6	0.651
Sex			**0.011**			0.110
Male	128 (30.8%)	106 (40.3%)		69 (31.5%)	86 (39.3%)	
Female	288 (69.2%)	157 (59.7%)		150 (68.5%)	133 (60.7%)	
BMI (kg/m^2^), mean (SD)	23.2 ± 3.7	23.1 ± 3.8	0.784	23.2 ± 3.5	23.2 ± 3.9	0.817
GLU (mmol/L), median (IQR)	5.2 (4.7–5.7)	5.1 (4.6–5.6)	0.662	5.3 (4.6–5.8)	5.0 (4.6–5.6)	0.356
ALB (g/L), median (IQR)	42.0 (39.0–45.0)	40.0 (37.0–43.0)	**< 0.001**	41.0 (38.0–44.0)	40.0 (38.0–43.0)	0.199
Abdominal surgery history	35 (8.4%)	42 (16.0%)	0.003	27 (11.8%)	29 (13.9%)	0.568
ASA score			**< 0.001**			0.116
1	330 (79.3%)	152 (57.8%)		157 (71.7%)	137 (62.6%)	
2	76 (18.3%)	95 (36.1%)		53 (24.2%)	72 (32.9%)	
3	10 (2.4%)	16 (6.1%)		9 (4.1%)	10 (4.6%)	
CA 19-9			0.053			0.463
≤ 35 IU/L	385 (94.8%)	224 (90.7%)		205 (93.6%)	201 (91.8%)	
> 35 IU/L	21 (5.2%)	23 (9.3%)		14 (6.4%)	18 (8.2%)	
PV/SMV abutment	7 (1.7%)	6 (2.3%)	0.579	4 (1.8%)	5 (2.3%)	1.000
Tumor size (cm), median (IQR)	2.8 (2.0–4.5)	4.0 (2.5–5.6)	**< 0.001**	2.5 (2.0–5.0)	3.5 (2.2–5.0)	0.154
Pathology			**0.010**			0.889
Cystic neoplasm	192 (46.2%)	96 (36.5%)		88 (40.2%)	79 (36.1%)	
Solid neoplasm (SPT and PNET)	136 (32.7%)	82 (31.2%)		68 (31.1%)	74 (33.8%)	
IPMN	40 (9.6%)	32 (12.2%)		24 (11.0%)	27 (12.3%)	
Inflammatory neoplasm	27 (6.5%)	34 (12.9%)		23 (10.5%)	25 (11.4%)	
Others^a^	21 (5.1%)	19 (7.2%)		16 (7.3%)	14 (6.4%)	
Tumor location			0.169			0.410
Tail	172 (41.4%)	107 (40.7%)		98 (44.7%)	94 (42.9%)	
Body–tail junction	91 (21.9%)	44 (16.7%)		41 (18.7%)	33 (15.1%)	
Body and neck	153 (36.8%)	112 (42.6%)		80 (36.5%)	92 (42.0%)	

*P* values < 0.05 indicate a significant difference between the two groups are given in bold

Others^a^: including teratoma, mesothelioma, angioma

### Perioperative outcomes

There were three cases (0.7%) of conversion to laparotomy in the unmatched RDP group. The reasons for conversion in the RDP group included two cases of severe abdominal adhesion and 1 case of uncontrollable intraoperative bleeding from the splenic vein. No vascular resection and reconstruction occurred.

After matching, the RDP cohort had a significantly shorter operative duration (120 min vs. 180 min, *P* < 0.001) and less estimated blood loss (50 ml vs. 200 ml, *P* < 0.001) than the ODP cohort. The RDP and ODP cohorts showed similar rates of R0 resection (95.4% vs 97.3%, *P* = 0.445), while the RDP cohort showed a significantly higher spleen preservation rate (63.5% vs 26.5%, *P* < 0.001). In terms of postoperative complications, the two cohorts showed no significant difference in the incidence of POPF, DGE, or PPH, while the RDP cohort showed a lower infection rate (4.6% vs 12.3%, *P* = 0.006) and earlier gastrointestinal function recovery [3 (2–4) vs 3 (3–5), *P* = 0.019]. The number of days to oral intake, severe complications with a Clavien–Dindo score ≥ 3, 90-day mortality rate and postoperative LOS also showed equivalence between the two cohorts (Table 2).

**Table 2:** Intraoperative and perioperative characteristics of the population before and after matching

	Before propensity score matching			After propensity score matching		
	RDP (*N* = 416)	ODP (*N* = 263)	*P* value	RDP (*N* = 219)	ODP (*N* = 219)	*P* value
Conversion to laparotomy	3	/	/	1	/	/
Operative time (min), median (IQR)	120 (90–150)	180 (130–210)	**< 0.001**	120 (90–150)	175 (130–210)	**< 0.001**
Estimated blood loss (ml), median (IQR)	50 (30–150)	200 (100–400)	** < 0.001**	50 (30–175)	200 (100–300)	** < 0.001**
R0 resection, *n* (%)	402 (96.6%)	256 (97.3%)	0.606	209 (95.4%)	213 (97.3%)	0.445
Spleen preservation, *n* (%)	277 (66.6%)	65 (24.7%)	**< 0.001**	139 (63.5%)	58 (26.5%)	**< 0.001**
Spleen preservation method, *n* (%)			**< 0.001**			**< 0.001**
Splenectomy	139 (33.4%)	198 (75.3%)		80 (36.5%)	161 (73.5%)	
KT	202 (48.6%)	39 (14.8%)		101 (46.1%)	35 (16.0%)	
WT	75 (18.0%)	26 (9.9%)		38 (17.4%)	23 (10.5%)	
POPF, *n* (%)	93 (22.4%)	82 (31.2%)	**0.010**	53 (24.2%)	70 (32.0%)	0.071
CR- POPF, *n* (%)	53 (12.7%)	47 (17.9%)	0.066	32 (14.6%)	41 (18.7%)	0.249
DGE, *n* (%)	6 (1.4%)	5 (1.9%)	0.758	5 (2.3%)	5 (2.3%)	1.000
Infection, *n* (%)	20 (4.8%)	33 (12.6%)	**< 0.001**	10 (4.6%)	27 (12.3%)	**0.006**
PPH, *n* (%)	10 (2.4%)	6 (2.3%)	0.918	8 (3.7%)	5 (2.3%)	0.573
Others^a^, *n* (%)	10 (2.4%)	9 (3.4%)	0.433	7 (3.2%)	6 (2.7%)	1.000
Reoperation, *n* (%)	11 (2.6%)	7 (2.7%)	0.989	8 (3.7%)	7 (3.2%)	1.000
Clavien–Dindo ≥ 3	12 (2.9%)	10 (3.8%)	0.511	9 (4.1%)	8 (3.7%)	1.000
90-day mortality, *n* (%)	1 (0.2%)	2 (0.8%)	0.563	1 (0.5%)	1 (0.5%)	1.000
Readmission, *n* (%)	11 (2.6%)	14 (5.3%)	0.071	7 (3.2%)	11 (5.0%)	0.470
GI function, days, median (IQR)	3 (2–4)	4 (3–5)	**< 0.001**	3 (2–4)	3 (3–5)	**0.019**
Oral intake, days, median (IQR)	4.5 (3–7)	3 (2–5)	**0.005**	5 (3–7)	3 (2–5)	**0.006**
LOS (days), median (IQR)	13 (11–19)	16 (12–21)	**0.029**	14 (11–19)	16 (11.5–20)	0.407

*P* values < 0.05 indicate a significant difference between the two groups are given in bold

*RDP* robotic-assisted distal pancreatectomy, *ODP* open distal pancreatectomy, *KT* Kimura technique, *WT* Warshaw technique, *POPF* postoperative pancreatic fistula, *CR-POPF* clinically relevant POPF (ISGPF grade B and C), *PPH* post pancreatectomy hemorrhage; *DGE* delayed gastric emptying, *LOS* postoperative length of stay; others^a^: including pneumonia, deep vein thrombosis, heart disease, cerebral hemorrhage, pleural effusion

In logistic regression analysis for predicting DP with splenectomy (failure of spleen preservation) and splenic vessel sacrifice (failure of KT SPDP) in DP for benign and low-grade malignant tumors, the robotic approach, together with age, tumor size, pathological type of inflammatory neoplasm, and estimated blood loss, were independent predictors of DP with splenectomy. The robotic approach, together with age, elevated CA 19-9 level, tumor size, pathological type of an inflammatory neoplasm, tumor location in the pancreatic body and neck, and estimated blood loss, were found to be independent predictors of splenic vessel sacrifice (Tables 3, 4).

**Table 3:** Logistic regression analysis predicting splenectomy (failure of spleen preservation)

	Univariate analysis		Multivariate analysis	
	HR (95% CI)	*P* value	HR (95% CI)	*P* value
Approach				
ODP	Ref			
RDP	0.165 (0.117, 0.233)	**< 0.001**	0.239 (0.158, 0.361)	**< 0.001**
Age	1.014 (1.004, 1.024)	**0.008**	1.015 (1.001, 1.029)	**0.033**
Sex				
Male	Ref			
Female	0.696 (0.506, 0.956)	**0.025**	0.823 (0.537, 1.260)	0.370
BMI	0.984 (0.945, 1.024)	0.428		
ALB	0.956 (0.926, 0.988)	**0.007**	0.990 (0.948, 1.034)	0.647
Abdominal surgery history	1.047 (0.651, 1.683)	0.850		
ASA score				
1	Ref			
2	1.072 (0.756, 1.520)	0.695		
3	2.002 (0.875, 4.579)	0.100		
CA 19-9 (IU/L)				
≤ 35	Ref			
> 35	2.296 (1.194, 4.416)	**0.013**	1.696 (0.785, 3.666)	0.179
PV/SMV abutment	3.456 (0.943,12.669)	0.061		
Tumor size	1.283 (1.192, 1.381)	**< 0.001**	1.283 (1.173, 1.404)	**< 0.001**
Pathology				
Cystic neoplasm	Ref			
Solid neoplasm (SPT and PNET)	1.312 (0.921, 1.868)	0.133	1.634 (1.043, 2.560)	0.032
IPMN	1.608 (0.957, 2.703)	0.073	1.565 (0.827, 2.962)	0.169
Inflammatory neoplasm	6.939 (3.388,14.214)	**< 0.001**	5.121 (2.220, 11.811)	**< 0.001**
Others	1.113 (0.572, 2.165)	0.752	0.884 (0.386, 2.027)	0.771
Tumor location				
Tail	Ref			
Body–tail junction	1.051 (0.697, 1.585)	0.813		
Body and neck	1.108 (0.791, 1.551)	0.551		
Operative time	1.008 (1.005, 1.011)	**< 0.001**	0.998 (0.994, 1.002)	0.245
Estimated blood loss	1.004 (1.003, 1.005)	**< 0.001**	1.003 (1.001, 1.004)	**< 0.001**

*P* values < 0.05 indicate a significant difference between the two groups are given in bold

**Table 4:** Logistic regression analysis predicting Warshaw SPDP and splenectomy (failure of Kimura SPDP)

	Univariate analysis		Multivariate analysis	
	HR (95% CI)	*P* value	HR (95% CI)	*P* value
Approach				
ODP	Ref			
RDP	0.184 (0.125, 0.273)	**< 0.001**	0.265 (0.165, 0.425)	**< 0.001**
Age	1.011 (1.000, 1.021)	**0.044**	1.017 (1.002, 1.031)	**0.022**
Sex				
Male	Ref			
Female	0.840 (0.602, 1.173)	0.307		
BMI	0.968 (0.928, 1.009)	0.125		
ALB	0.980 (0.948, 1.012)	0.223		
Abdominal surgery history	1.090 (0.660, 1.798)	0.737		
ASA score				
1	Ref			
2	1.064 (0.739, 1.532)	0.739		
3	2.416 (0.895, 6.519)	0.082		
CA 19-9 (IU/L)				
≤ 35	Ref			
> 35	3.054 (1.339, 6.965)	**0.008**	2.548 (1.015, 6.393)	**0.046**
PV/SMV abutment	3,265,018.587 (0.000, Inf)	0.970		
Tumor size	1.439 (1.308, 1.584)	**< 0.001**	1.443 (1.289, 1.614)	**< 0.001**
Pathology				
Cystic neoplasm	Ref			
Solid neoplasm (SPT and PNET)	0.921 (0.642, 1.322)	0.655	1.268 (0.812, 1.982)	0.297
IPMN	1.200 (0.696, 2.070)	0.512	1.303 (0.681, 2.492)	0.424
Inflammatory neoplasm	5.500 (2.291, 13.203)	**< 0.001**	4.477 (1.692, 11.845)	**0.003**
Others	0.812 (0.415, 1.588)	0.542	0.913 (0.402, 2.074)	0.827
Tumor location				
Tail	Ref			
Body–tail junction	1.237 (0.807, 1.897)	0.329	1.487 (0.886, 2.494)	0.133
Body and neck	1.449 (1.018, 2.063)	**0.040**	1.692 (1.099, 2.607)	**0.017**
Operative time	1.008 (1.005, 1.011)	**< 0.001**	0.999 (0.995, 1.003)	0.554
Estimated blood loss	1.004 (1.003, 1.006)	**< 0.001**	1.003 (1.001, 1.004)	**0.001**

*P* values < 0.05 indicate a significant difference between the two groups are given in bold

## Discussion

A minimally invasive approach is considered technically available, safe and feasible for DP in the treatment of left-side pancreatic tumors [5, 26–29]. As an inevitable part of minimally invasive DP, RDP has been frequently analyzed in observational studies, and a series of reports have indicated that RDP results in a shorter operative duration, less blood loss, shorter postoperative hospital stay, higher spleen preservation rate, and comparable morbidity and mortality rates [30–34].

For patients with benign and low-grade malignant pancreatic tumors, good cosmetic effects are required, and low morbidity and organ preservation should be achieved. To compare the perioperative outcomes of RDP and ODP for such benign and low-grade malignant tumors in the pancreatic body/tail, we performed a retrospective analysis using a prospective database. In our study, we collected data from patients with benign and low-grade malignant tumors treated with RDP after the learning curve had been passed for comparison with data from patients treated with ODP during the same time period. PSM has been a popular statistical method in recent years to eliminate bias in observational studies [35], and PSM was applied in our study to eliminate selection bias caused by baseline population and tumor characteristics, such as age, sex, preoperative ALB level, abdominal surgery history, tumor size, and pathological type. After matching, the equivalence of these baseline indices made the results of the subsequent analysis more robust. A total of 416 cases of RDP and 263 cases of ODP were collected. After 1:1 matching, 219 cases in each group were matched and compared. We found that RDP had advantages in terms of operative duration, estimated blood loss, spleen preservation rate, KT SPDP rate, postoperative surgical site infection rate, and gastrointestinal function recovery. However, postoperative oral intake occurred later in the RDP cohort; this may have been influenced by the different protocols used to determine the postoperative oral intake time between the two cohorts. Altogether, these results reveal that RDP causes less surgical trauma and provides a better foundation for postoperative recovery. The robotic approach has been considered a particularly suitable approach for operations that emphasize meticulous and bloodless dissection, thus potentially expanding the indications for RDP [36, 37]. Studies focusing on RDP and benign and low-grade malignant tumors were separately analyzed, and this series of studies also showed that RDP had noninferior perioperative outcomes when compared with corresponding laparoscopic or open surgery in the treatment of these nonmalignant tumors [33, 38, 39]. These conclusions are in agreement with our results showing that RDP offers acceptable perioperative outcomes for benign and low-grade malignant pancreatic body/tail tumors.

In Guerrini’s study, RDP showed advantages over LDP in terms of the conversion rate (8.2 vs. 21.6%) [40]. In benign cases, a lower conversion rate indicates a better cosmetic effect. In our study, there were 3 cases (0.7%) of conversion to laparotomy in the RDP group, and severe abdominal adhesion and uncontrollable intraoperative bleeding led to conversion in all intraductal papillary mucinous neoplasia (IPMN) cases. In all the benign and low-grade malignant tumor cases, the conversion was always caused by IPMN and inflammatory neoplasms, and the precise dissection and suturing of the robotic system consistently provided better management in potential conversion cases. Moreover, the robotic approach always achieved good cosmetic effects.

SPDP, as an important technique in organ-preserving surgery for pancreatic body/tail nonmalignant tumors, has both hematological and immunological advantages, as proven by a series of studies [11, 12, 41, 42]. The WT is a relatively easy spleen-preserving method, and the spleen can often be vascularized by short gastric vessels and the left gastroepiploic artery. The feasibility and acceptable short-term outcomes of WT SPDP have also been proven in different studies [43, 44]. However, WT SPDP is associated with the risk of splenic infarction [45], and there are still some concerns about the long-term outcomes of the WT, such as gastric varices and the risk of gastric bleeding [46]. KT SPDP is the first choice and preferred method for pancreatic body/tail tumors with spleen preservation potential. After PSM to minimize selection bias, the RDP cohort showed a significantly higher spleen preservation rate and a higher rate of KT SPDP. In the RDP and ODP cohorts, the approaches for pancreatic parenchyma mobilization showed different trends, with a “bottom-up” view in RDP, which was differed from the “top-down” view in ODP. In our clinical practice, the intraoperative bleeding that causes the failure of KT SPDP is often caused by splenic vein bleeding when separating the pancreatic parenchyma from the splenic vessels. Using the robotic surgical system, the 3D view, EndoWrist and tremor elimination all helped to provide more delicate manipulation, precise dissection of the splenic vessels and more meticulous ligation or suturing the small branches of the splenic vessels, which may allow the difficulties in laparoscopic DP with spleen-preserving intention to be better overcome [19]. The advantages of SPDP were reconfirmed by the univariate and multivariate analyses. Age, tumor size, a higher proportion of inflammatory neoplasms and blood loss were independent risk factors of the spleen preservation rate and the KT SPDP rate. Larger tumor size and the nature of the inflammatory neoplasms were often associated with more difficult manipulation of the splenic vessels, and blood loss was often associated with surgical trauma and reflected the difficulty of the procedures. An elevated CA 19-9 level was identified in 44 cases in our study and was found to be an independent predictor of splenic vessel sacrifice (WT SPDP and DP with splenectomy). Elevated CA 19-9 levels have been reported to be associated with acute or chronic pancreatitis even in benign pancreatic tumors [47]. The inflammation caused by some types of benign and low-grade malignant tumors, such as inflammatory neoplasms and IPMN, may increase the level of CA 19-9 and ultimately cause the failure of splenic vessel preservation, which may explain why an elevated CA 19-9 level was an independent risk factor for KT SPDP.

The low morbidity rate, especially the lower rate of severe complications (Clavien–Dindo score ≥ 3), is also crucial in benign and low-grade malignant pancreatic tumors. POPF, as the most common postoperative complication, may cause subsequent intraoperative infection and PPH. The overall POPF rate was 24.2% in the RDP group vs 32.0% in the ODP group, and the corresponding clinically relevant POPF rates (grade B and C) were 14.6% vs 18.7%, respectively, both without a significant difference. In our study, we transected the pancreas with a stapler in most cases; thus, we did not need to address the pancreatic stump. In other cases in which we transected the pancreas with a harmonic scalpel, we always found the pancreatic duct and ligated it with sutures; the robotic system provided an enlarged view and allowed delicate manipulation, which facilitated handling of the pancreatic stump and reduced the POPF rate. The POPF rate in our study is comparable to that in other series of RDP, such as the rate of 17% reported by Gavriilidis in a systematic review and network meta-analysis [48]. PPH, which is a severe complication after DP, always requires reoperation (including intervention). In our initial practice, before we had mastered this approach, massive PPH with hypovolemic shock occurred in three cases of RDP with the KT, and reoperation was performed (including digital subtraction angiography, celiac artery angiography and splenic artery embolization). After passing the learning curve, we performed more precise manipulations of the small branches of the pancreatic transverse and dorsal arteries, and massive PPH seldom occurred.

There were several limitations to this study, which should be noted. Although the PSM method was applied to reduce selection bias, the retrospective nature of the study cannot be ignored. In addition, despite preoperative MDT discussion and performing the operations based on the intention-to-treat principle, some subjective factors still influenced the intraoperative decision, such as inflammation, adhesions and difficulty in mobilizing the splenic vessels, which may influence the surgeon’s choice of the KT, the WT or splenectomy. Third, the follow-up data were missing in some cases, which prevented further analysis of the long-term outcomes between the RDP and ODP groups. Finally, as an irreplaceable minimally invasive pancreatic surgery, LDP also plays an important role in patient treatment, but in our department, since the introduction of the da Vinci robotic system in 2010, many LDP surgeries for benign and low-grade malignant tumors have been replaced by RDP according to our initial experience and that of other surgeons [31, 45]. Therefore, a lack of sufficient clinical data for LDP prevents subsequent comparisons among robotic, laparoscopic and open approaches for DP.

## Conclusions

For benign and low-grade malignant tumors in the pancreatic body/tail, the perioperative outcomes of RDP were better than those of ODP in terms of the operative duration, estimated blood loss, spleen preservation, infection rate and gastrointestinal function recovery. The robotic approach was an independent predictor of both spleen preservation and KT SPDP. However, this was a retrospective study with inherent drawbacks. Although we used the PSM method to minimize bias, further randomized controlled studies should be designed to verify the value of RDP for benign and low-grade malignant tumors.

## Electronic supplementary material

Below is the link to the electronic supplementary material.

Supplementary file1 (DOCX 13 kb)

Supplementary file2 (DOCX 14 kb)
