# Supplementary material for: Robotic-assisted versus open distal pancreatectomy for benign and low-grade malignant pancreatic tumors: a propensity score-matched study
Source: Surg Endosc. 2020 Aug 11;35(5):2255–64. doi: 10.1007/s00464-020-07639-9 (PMC8057962; doi:10.1007/s00464-020-07639-9)
Supplement: Supplementary file 1 — Supplementary file1 (DOCX 13 kb) [file 464_2020_7639_MOESM1_ESM.docx]

**Supplementary Table 1: Perioperative characteristics between SPDP and DP with splenectomy after matching**

|  | SPDP (N=197) | DP with splenectomy (N= 241) | P value |
| --- | --- | --- | --- |
| Operative time, min, median (IQR) | 120 (100-170) | 150(120-200) | **<0.001** |
| Estimated blood loss, ml, median (IQR) | 100 (50-200) | 150 (100-300) | **<0.001** |
| R0 resection, n (%) | 189 (95.9%) | 233 (96.7%) | 0.681 |
| POPF, n (%) | 59 (29.9%) | 64 (26.6%) | 0.432 |
| CR- POPF, n (%) | 32 (14.6%) | 41 (18.7%) | 0.830 |
| DGE, n (%) | 1 (0.5%) | 9 (3.7%) | **0.026** |
| Infection, n (%) | 14 (7.1%) | 23 (9.5%) | 0.362 |
| PPH, n (%) | 7 (3.6%) | 6 (2.5%) | 0.514 |
| Others*, n (%) | 3 (1.5%) | 10 (4.1%) | 0.107 |
| Reoperation, n (%) | 6 (3.0%) | 9 (3.7%) | 0.693 |
| Clavien-Dindo ≥3 | 7 (3.6%) | 10 (4.1%) | 0.748 |
| 90-day mortality, n (%) | 0 (0.0%) | 2 (0.8%) | 0.504 |
| Readmission, n (%) | 6 (3.0%) | 12 (5.0%) | 0.311 |
| GI function, days, median (IQR) | 3 (2-4) | 3 (2-5) | 0.142 |
| Oral intake, days, median (IQR) | 4 (2.5-7) | 4 (2-6) | 0.992 |
| LOS, days, median (IQR) | 13 (11-19) | 16 (12-20) | **0.003** |
