# Supplementary material for: Robotic-assisted versus open distal pancreatectomy for benign and low-grade malignant pancreatic tumors: a propensity score-matched study
Source: Surg Endosc. 2020 Aug 11;35(5):2255–64. doi: 10.1007/s00464-020-07639-9 (PMC8057962; doi:10.1007/s00464-020-07639-9)
Supplement: Supplementary file 2 — Supplementary file2 (DOCX 14 kb) [file 464_2020_7639_MOESM2_ESM.docx]

**Supplementary Table 2: Perioperative characteristics between Kimura SPDP and Warshaw SPDP after matching**

|  | Kimura (N=136) | Warshaw (N=61) | P value |
| --- | --- | --- | --- |
| Operative time, min, median (IQR) | 120 (100-157.5) | 140(115-180) | 0.061 |
| Estimated blood loss, ml, median (IQR) | 100 (30-200) | 100 (50-200) | **0.036** |
| R0 resection, n (%) | 130 (95.6%) | 59 (96.7%) | 0.710 |
| POPF, n (%) | 41 (30.1%) | 18 (29.5%) | 0.928 |
| CR- POPF, n (%) | 24 (17.6%) | 8 (13.1%) | 0.425 |
| DGE, n (%) | 1 (0.7%) | 0 (0.0%) | 1.000 |
| Infection, n (%) | 10 (7.4%) | 4 (6.6%) | 0.841 |
| PPH, n (%) | 5 (3.7%) | 2 (3.3%) | 0.889 |
| Others*, n (%) | 2 (1.5%) | 1 (1.6%) | 1.000 |
| Reoperation, n (%) | 4 (2.9%) | 2 (3.3%) | 0.899 |
| Clavien-Dindo ≥3 | 5 (3.7%) | 2 (3.3%) | 0.889 |
| 90-day mortality, n (%) | 0 (0.0%) | 0 (0.0%) | / |
| Readmission, n (%) | 5 (3.7%) | 1 (1.6%) | 0.442 |
| GI function, days, median (IQR) | 3 (2-4) | 3 (2-4) | 0.808 |
| Oral intake, days, median (IQR) | 3 (3-7) | 4 (2-7) | 0.761 |
| LOS, days, median (IQR) | 13 (11-19) | 13 (11-18) | 0.692 |
